# Supplementary material for: Deep FLASH-seq profiling of purified canine sensory neurons uncovers species-specific signatures relevant to pain and itch
Source: Pain. Author manuscript; Available in PMC 2026 Oct 1. (PMC7619458; doi:10.1097/j.pain.0000000000004102)
Supplement: Supplementary file 1 [file EMS218125-supplement-Supp_Material.pdf]

Supplemental Information for

Deep FLASH-seq profiling of purified canine sensory neurons uncovers species-specific signatures relevant to pain and itch

Paula Ledesma Fernandez[1,2], Brandi Butler[2], Heidi Theis[3], Stefan Paulusch[3], Elena De Domenico[3], Greg A Weir[1], Andrew M Bell[1,2].

[1]Spinal Cord Group, School of Psychology and Neuroscience, University of Glasgow, Glasgow G12 8QQ, United Kingdom; [2]School of Biodiversity, One Health and Veterinary Medicine, University of Glasgow, Glasgow G12 8QQ, United Kingdom; [3]Deutsches Zentrum für Neurodegenerative Erkrankungen (DZNE) e.V., PRECISE Platform for Genomics and Epigenomics at DZNE and University of Bonn, 53127 Bonn, Germany.

Contains Supplemental figures 1-10 & Tables S1, S2, S3, S5 & S6

Supplemental Figures

Fig S1. Confocal images from 4 animals showing the ratio of neurons to non-neuronal cells in cDRG sections (NeuN/DAPI staining).

Fig S2. RNA integrity scores, FACS gating strategy, pre- and post-sort cell suspensions, pilot plate GFAP cluster, and PCA of FACS parameters.

Fig S3. Quality control metrics for FLASH-seq data across plates, sexes, animals and sorting sessions, with cluster proportions and UMAPs after CCA integration.

Fig S4. Seurat clustering results at four different resolutions (res 1, 1.5, 2, 3); resolution 1.5 was used for the final dataset.

Fig S5. Expression of published cross-species DRG marker gene sets (Jung et al. 2023; Bhuiyan et al. 2024) across canine FLASH-seq clusters.

Fig S6. PCA and pairwise scatter plots of FACS parameters showing that large-diameter (NTRK3+) neurons cannot be distinguished from smaller neurons by FACS alone.

Fig S7. Convergent cross-species gene expression patterns for major functional gene families (ion channels, GPCRs, transcription factors, neuropeptides, synaptic proteins) across unified dog/human/mouse clusters.

Fig S8. Top 20 divergent genes between dog and human within each unified cluster.

Fig S9. Top 20 divergent genes between dog and mouse within each unified cluster.

Fig S10. Supporting data for ISH analyses: GFRA2 expression across species, Xenium human DRG in situ image, and bulk sequencing comparison of canine vs human DRG transcript abundance.

Supplemental Tables

Table S1. HCR™ Gold RNA-FISH probe and amplifier details for all ISH experiments.

Table S2. Neuronal diameter data (mean, median, SD, range) for each dog used in size analysis.

Table S3. Individual dog information: breed, sex, age, RIN score, and experimental use.

Table S4. Cross-species differential expression data (separate Excel file). Contains three sheets: convergent markers across dog/human/mouse; divergent markers between dog and human; divergent markers between dog and mouse. See Info sheet for full column definitions.

Table S5. Neuronal counts by size category (small/medium/large) for SSTR2 and KIT expression categories from ISH analysis.

Table S6. Full list of domestication positively selected genes overlapping with cluster DEGs, organised by cluster.

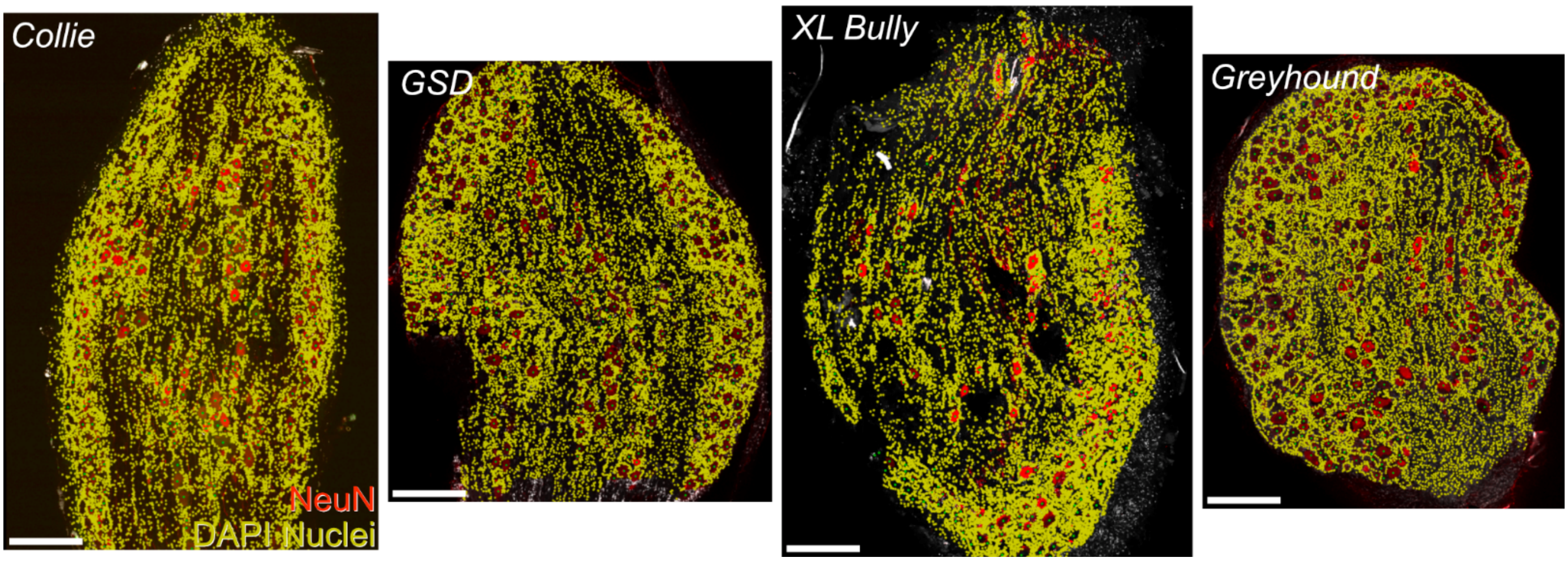

Fig S1. Example confocal images of cDRG from 4 animals in which the ratio of neurons to non-neuronal cells has been estimated. Neurons are stained with NeuN and all nuclei stained with DAPI. Each yellow dot annotation represents a single counted non-neuronal nucleus, while each green dot represents a counted neuron. Images are single confocal sections acquired at one airy unit with a 20x objective. Scale bars represent 400µm.

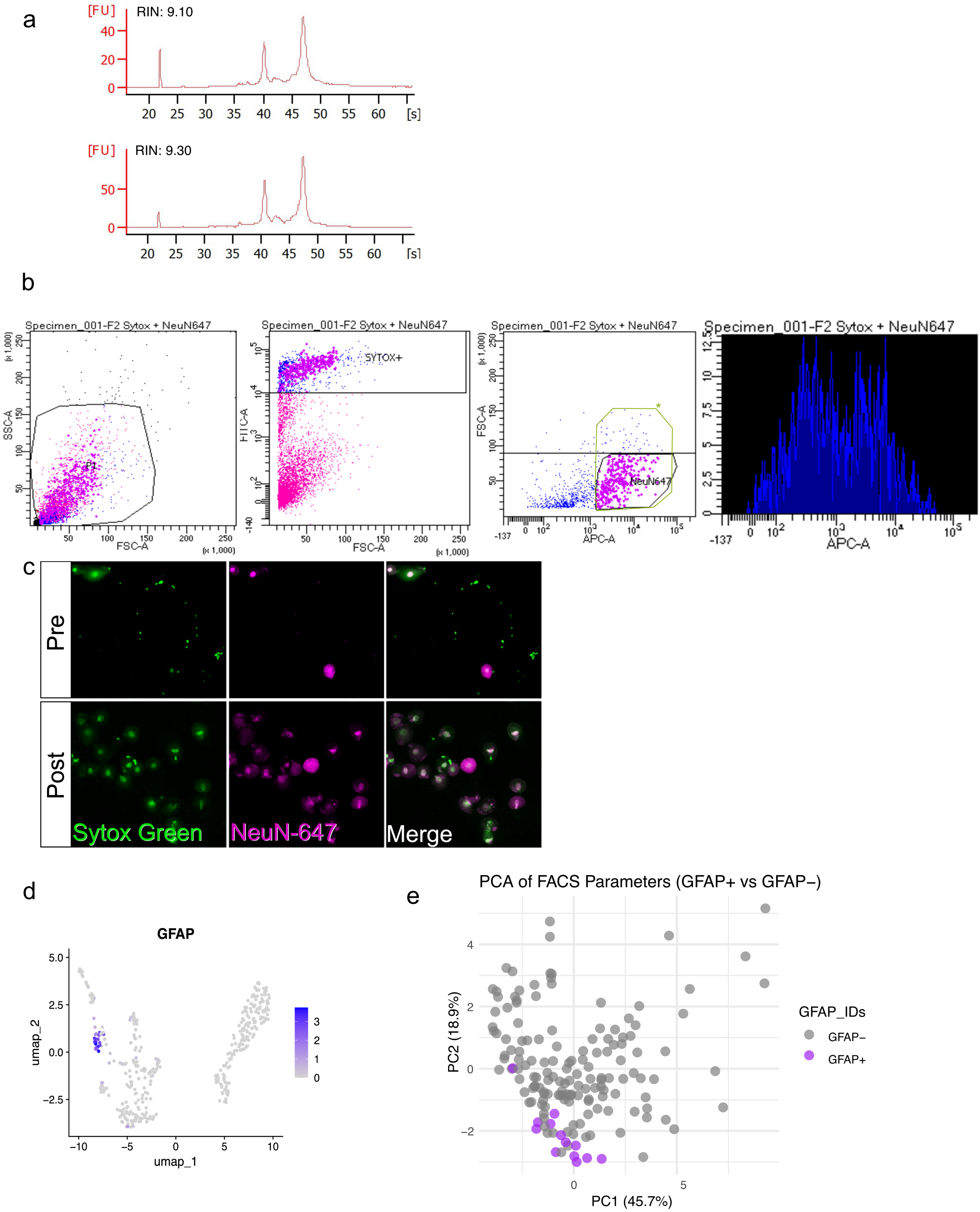

Fig S2. **(a)** Example RNA Integrity Scores (RIN) of DRG collected from dogs submitted for sequencing. **(b)** FACS gates shown in order (left to right) to achieve neuronal isolation for plate-sorting. SSC-A vs FSC-A gate scatter gate differentiated small debris from putative cells; FITC-A vs FSC-A gate was used to identify cells with high SytoxGreen fluorescence; FSC-A Vs APC-A gate identified NeuN647 positive cells

(neurons). Refinement of Neun647 gating was aided by 1-parameter histogram of the APC-A fluorescent channels, X axis is APC-A and Y axis is number of events (counts) for each fluorescent level. Events included after the inflection point (observed at 10^3 in this graph) were sorted into a plate. FSC-A threshold was informed by which FSC-A level filtered out complex cells formed by neurons with immune cells attached on its surface. To do this we isolated cells at different FSC-A levels followed by observation under the microscope. Notice that FSC-A upper threshold was reduced from 150000 (green square, *) to 100000 between trial and definitive plates due to high of number of well with no sequencing outcome resulting from events above this threshold. **(c) Top -Pre FACS cell suspension.** Representative fluorescence image after DRG dissociation but prior to FACS. Nuclei were labelled with SytoxGreen to visualise all nucleated events; NeuN647 staining highlights neuronal cells. Notice the heterogeneous mixture of neuronal cells and non-neuronal nuclei. **Bottom-Post FACS cell suspension**. Fluorescence image of the sorted population gated for SytoxGreen+/Neun647+ cells. Post-sort fractions show a visible enrichment for neuronal cells and preservation of neuronal cell integrity. **(d) Glial cells (GFAP+) cluster seen in Trial Plate FeaturePlot.** Presence of a distinguishable GFAP cluster evidenced minor glial cell contamination during FACS sorting. GFAP expression in glial cells, allows removal of this cluster for downstream analysis. **(e) PC1 Vs PC2 scatter plot from principal Component analysis (PCA) reveals that it is not possible to differentiate GFAP cells from neuronal cells through FACS parameters alone.** PCA analysis using all FACS parameters was performed to assess if GFAP cells were distinguishable from non-GFAP cells during FACS. Each purple dot in the scatter plot represents a GFAP-positive cell, and each grey dot corresponds to a non-GFAP cell. The first two principal components accounted for a substantial proportion of the total variance, PC1 explaining 45.7% and PC2 explaining 18.9% of the observed variability. However, the overlapping distributions indicates that the measured features included in the PCA are insufficient to reliably distinguish these two cell populations.

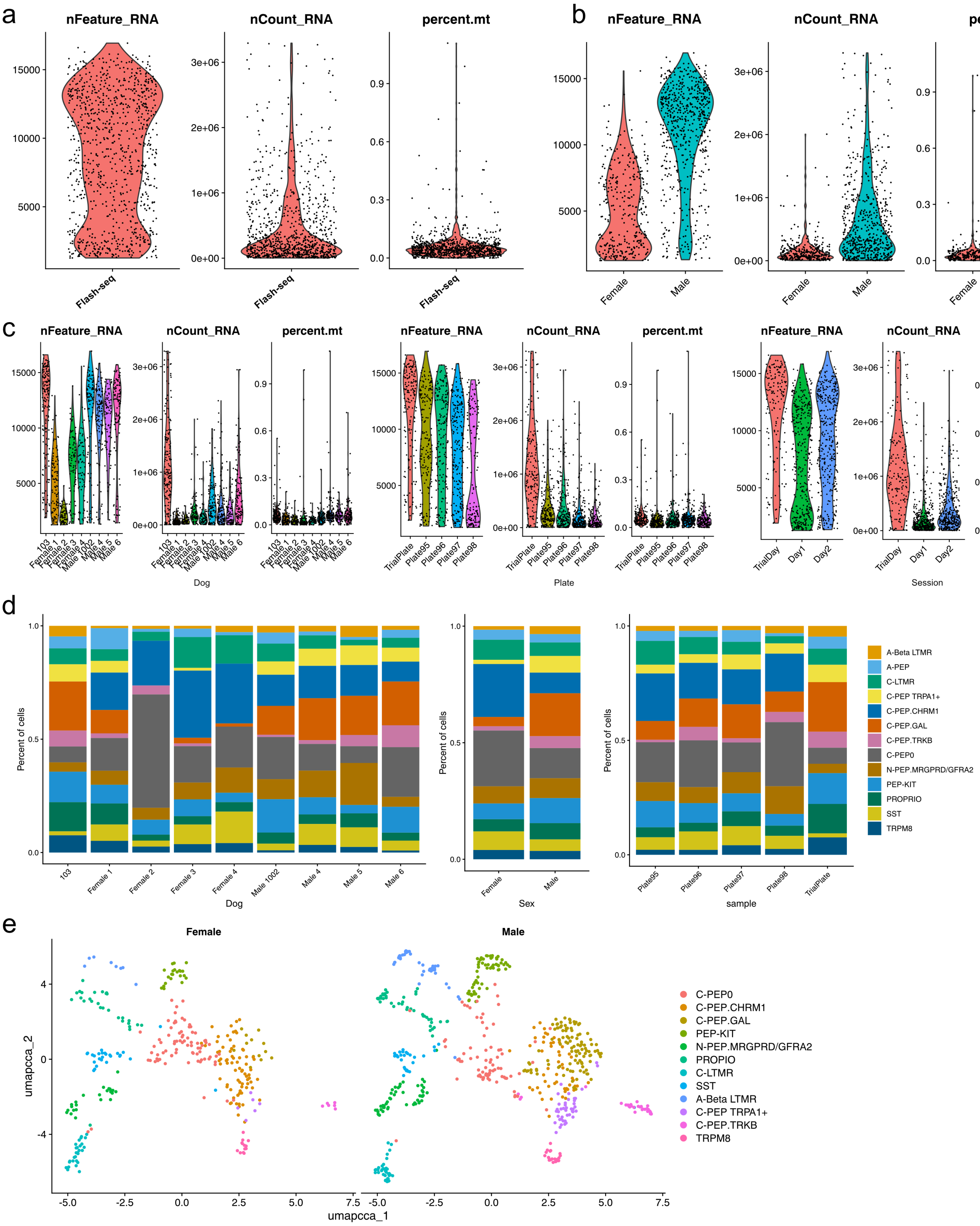

Fig S3. Quality control metrics for FLASH-seq sequencing data. (a) Violin plots showing number of unique genes per cell (nFeature_RNA), total per cell counts (nCount_RNA) and mitochondrial gene percentages (percent.mt). **(b&c)** Violin plots showing these same QC metrics across sexes, animals, plates and sorting sessions. **(d)** Stacked bar plots of final cluster proportions across animals, sexes and plates after CCA integration for batch correction. **(e)** UMAP plots showing final clusters separated by those seen in male and female animals after CCA integration.

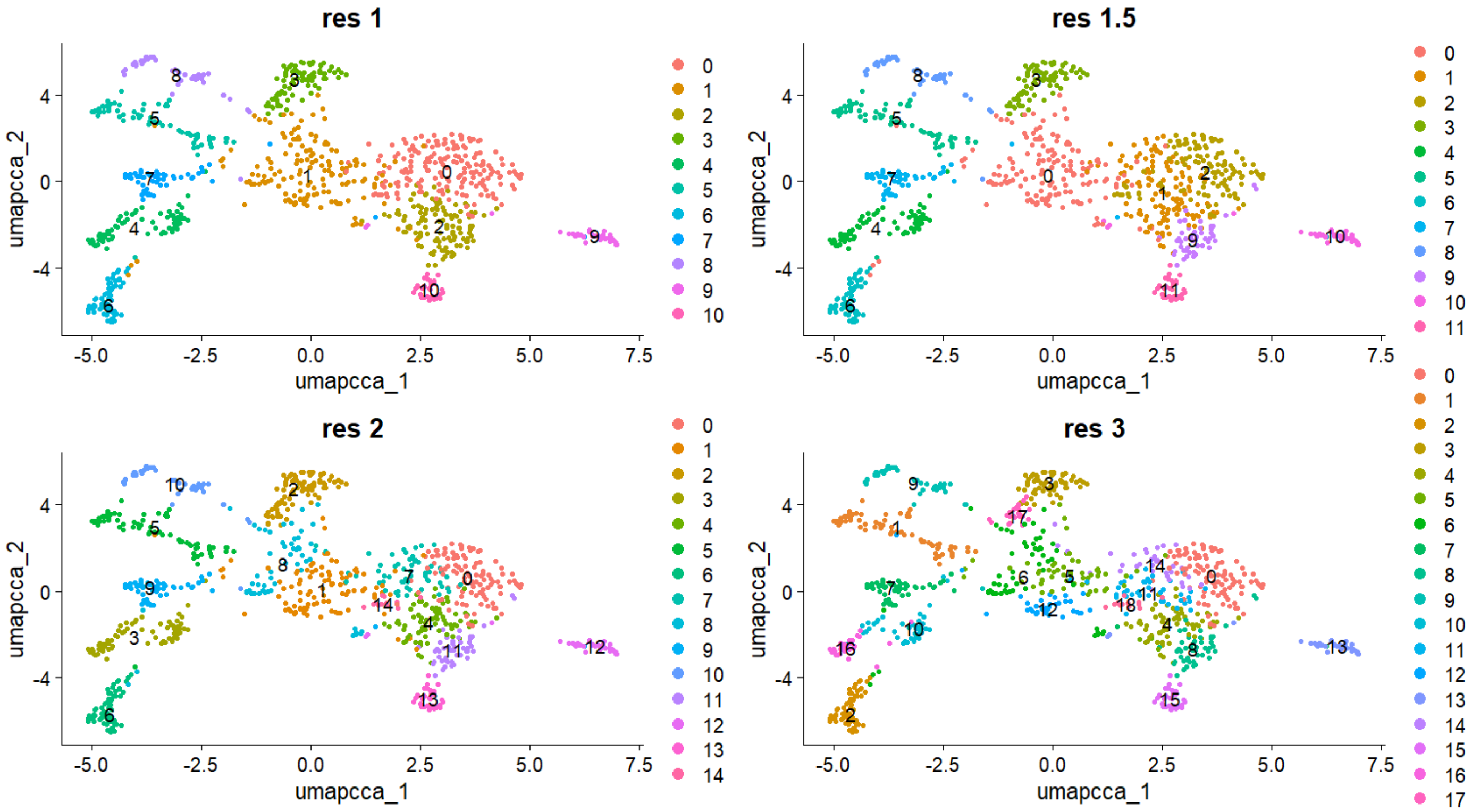

Fig S4. Results of different cluster resolutions on Seurat clustering. A resolution of 1.5 was used for the final dataset.

a. DRG Neuronal subtype marker genes from Jung et al. 2023

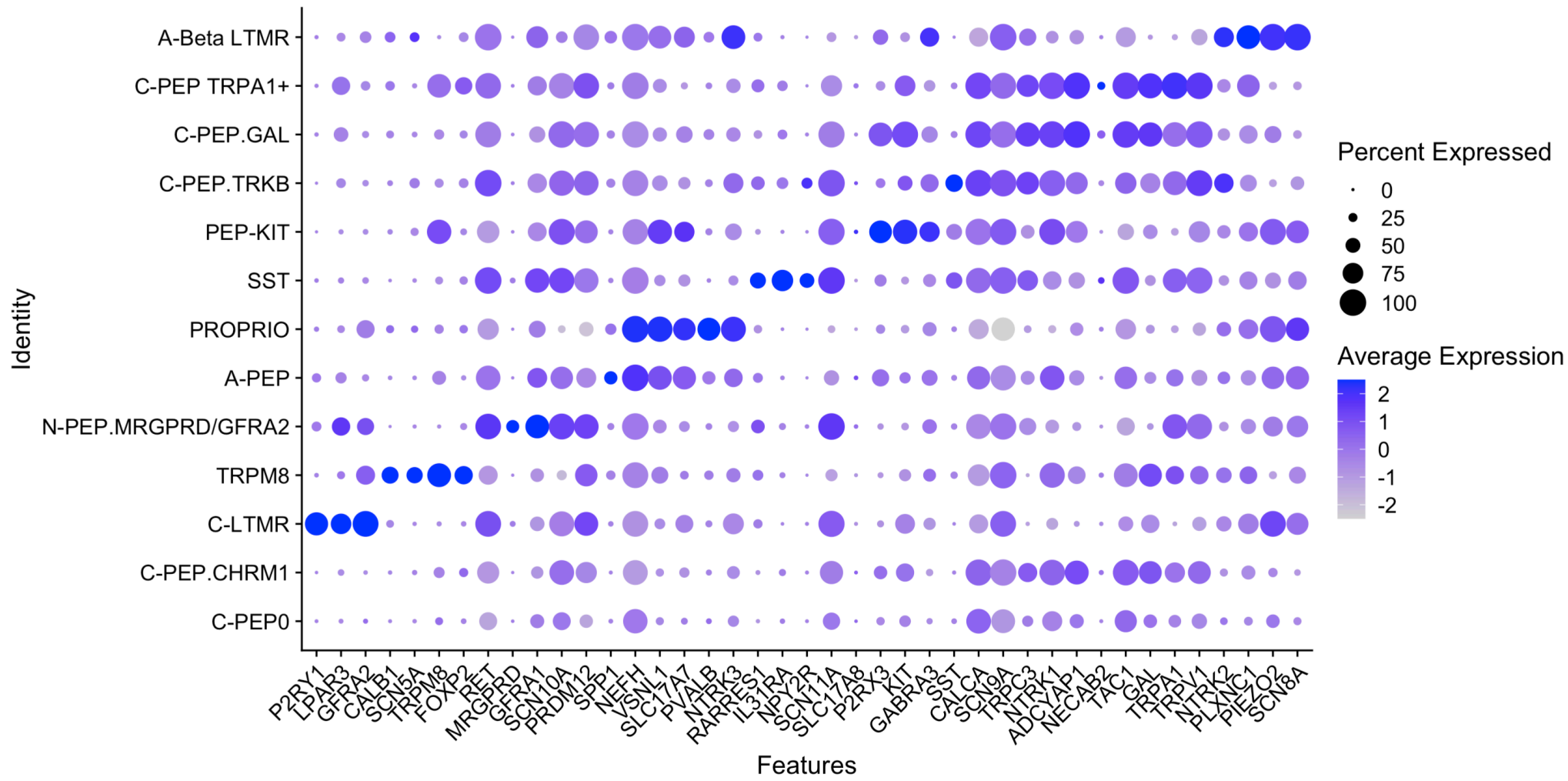

b. DRG Neuronal subtype marker genes from Bhuiyan et al. 2024

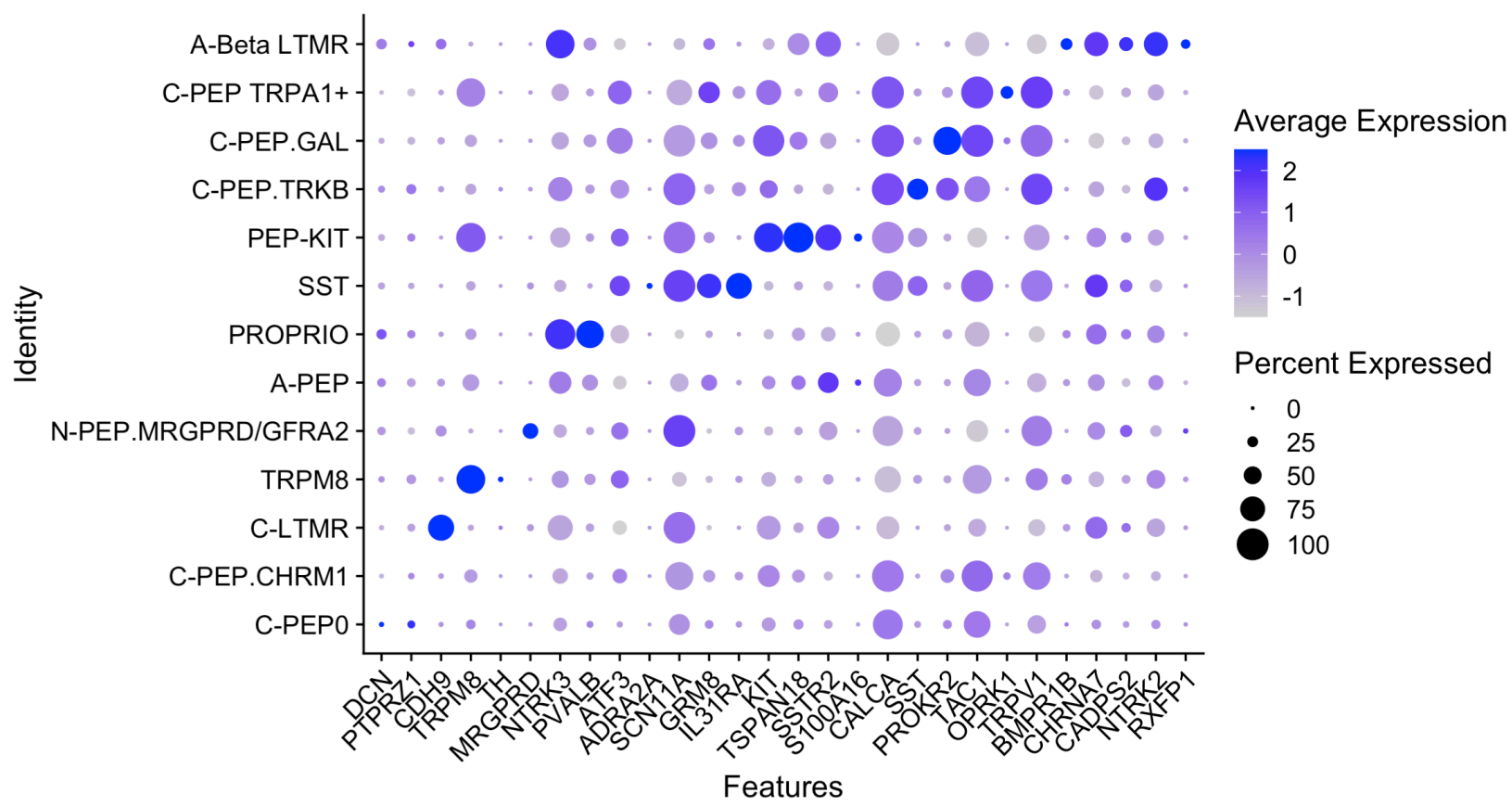

Fig S5. Marker genes from other cross-species atlases. Expression shown across canine FLASH-seq clusters. Dot colour represents scaled mean expression; dot size represents the percentage of expressing cells.

a

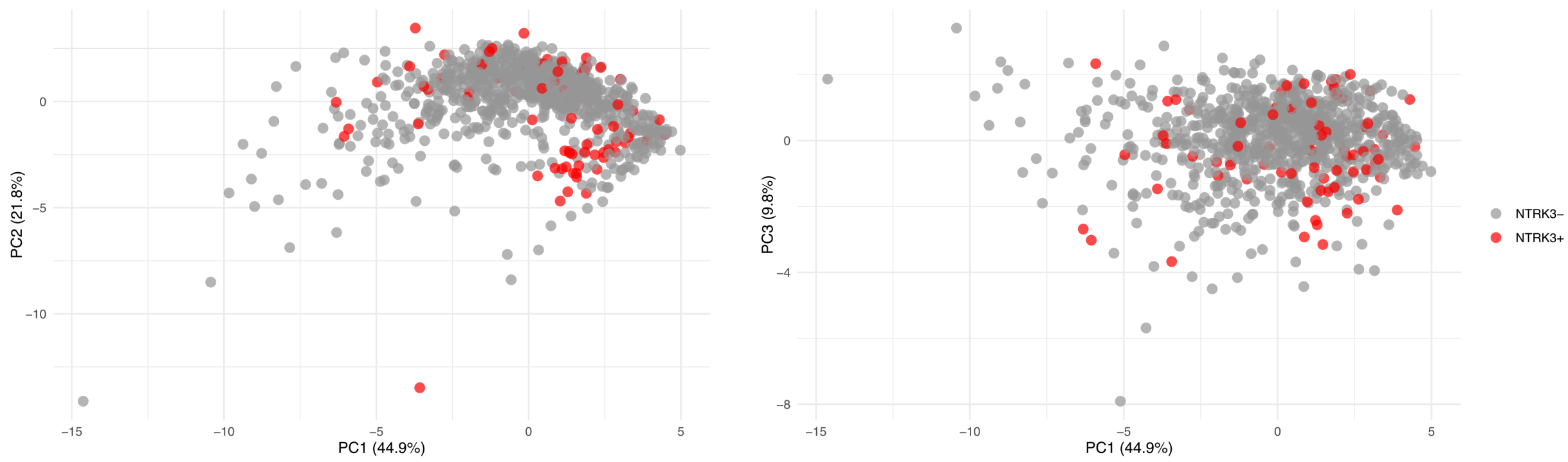

b

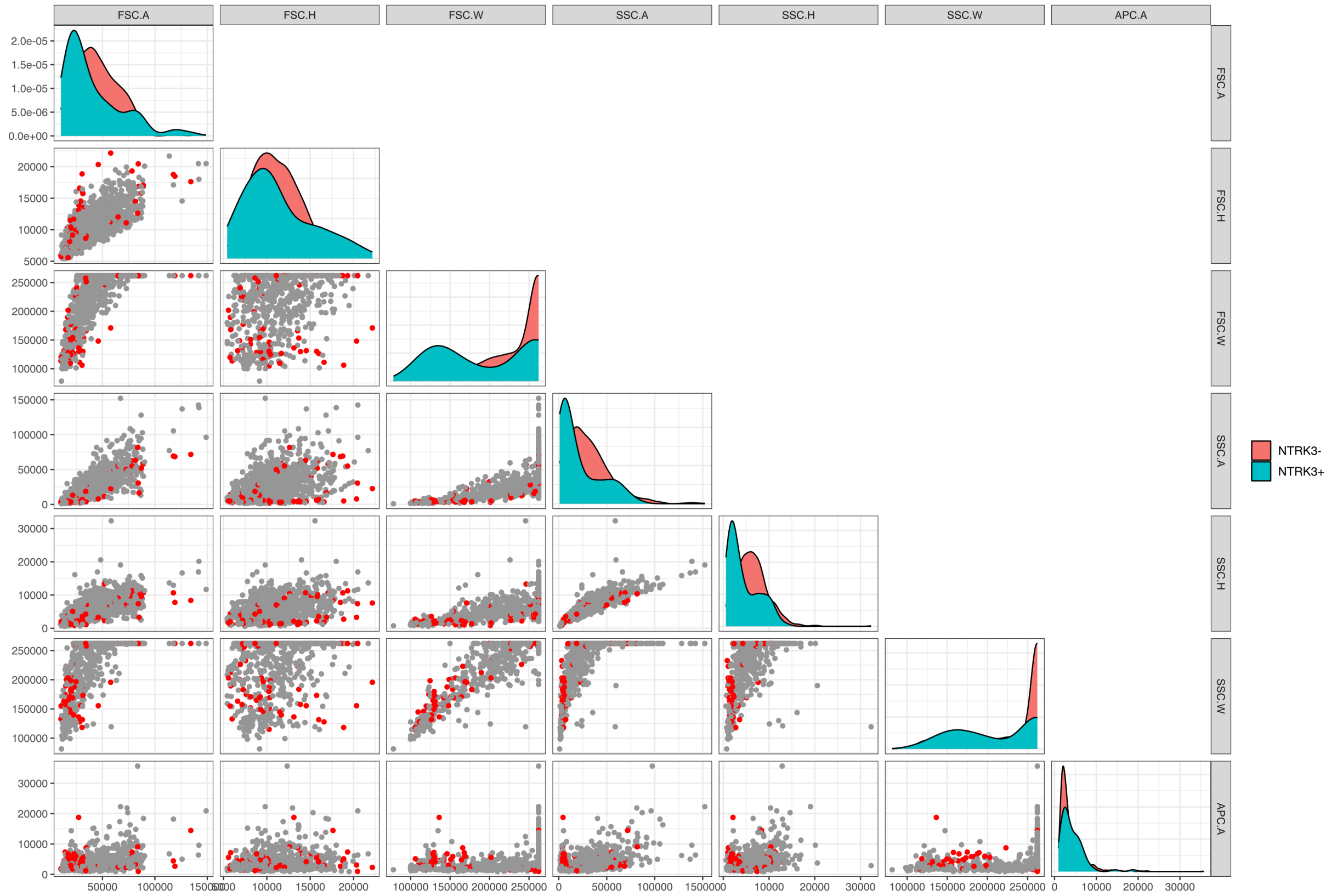

Fig S6. Non-distinguishable nature of large vs small diameter neurons using FACS parameters. (a) Principal component analysis of FACS parameters (scatter and fluorescence parameters) with large (NTRK3+) and other cells indicated by colour. Putative large cells overlap significantly in the principal component space, indicating that FACS parameters alone cannot be used to separate these cells. **(b)** Pairwise scatter plots of FACS scatter gating parameters, again with NTRK3+ cells indicated in red. Although large cells appear to show reduced scatter, they are not separable from other cells.

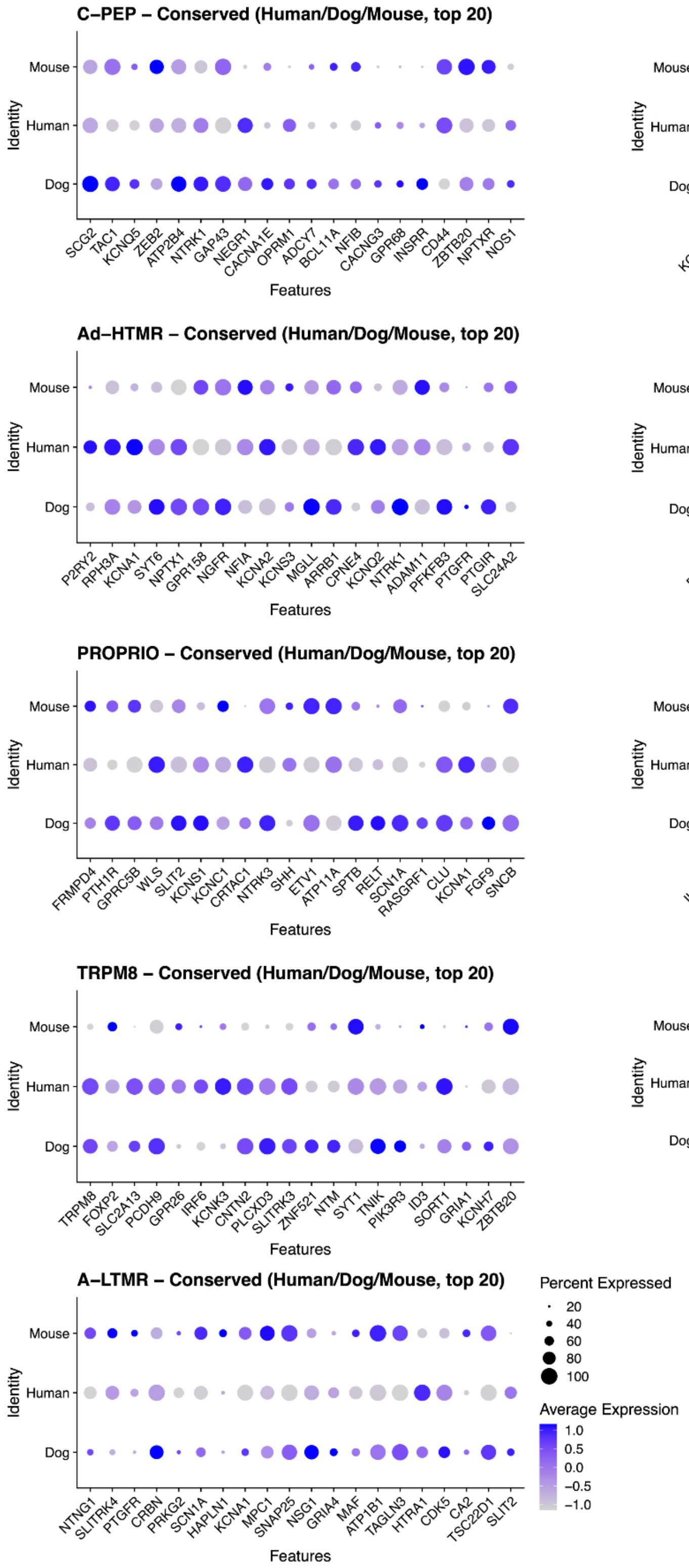

Fig S7. Cross-species convergent expression patterns of functional genes across unified neuronal clusters. Dot plots showing expression of all genes belonging to major functional categories (transcription factors, GPCRs, ligands, ion channels, synaptic proteins) across the unified dog, human, and mouse DRG clusters. Dot colour represents scaled mean expression; dot size represents the percentage of expressing cells. For each broad class of sensory neuron, the top 20 convergent genes are shown. These were identified using FindConservedMarkers, retaining only genes significantly enriched in that cluster in all three species ($\log2FC \geq 0.25$, $\geq 10\%$ expressing, adjusted $P < 0.05$) and ranking them by the maximum adjusted P value across species.

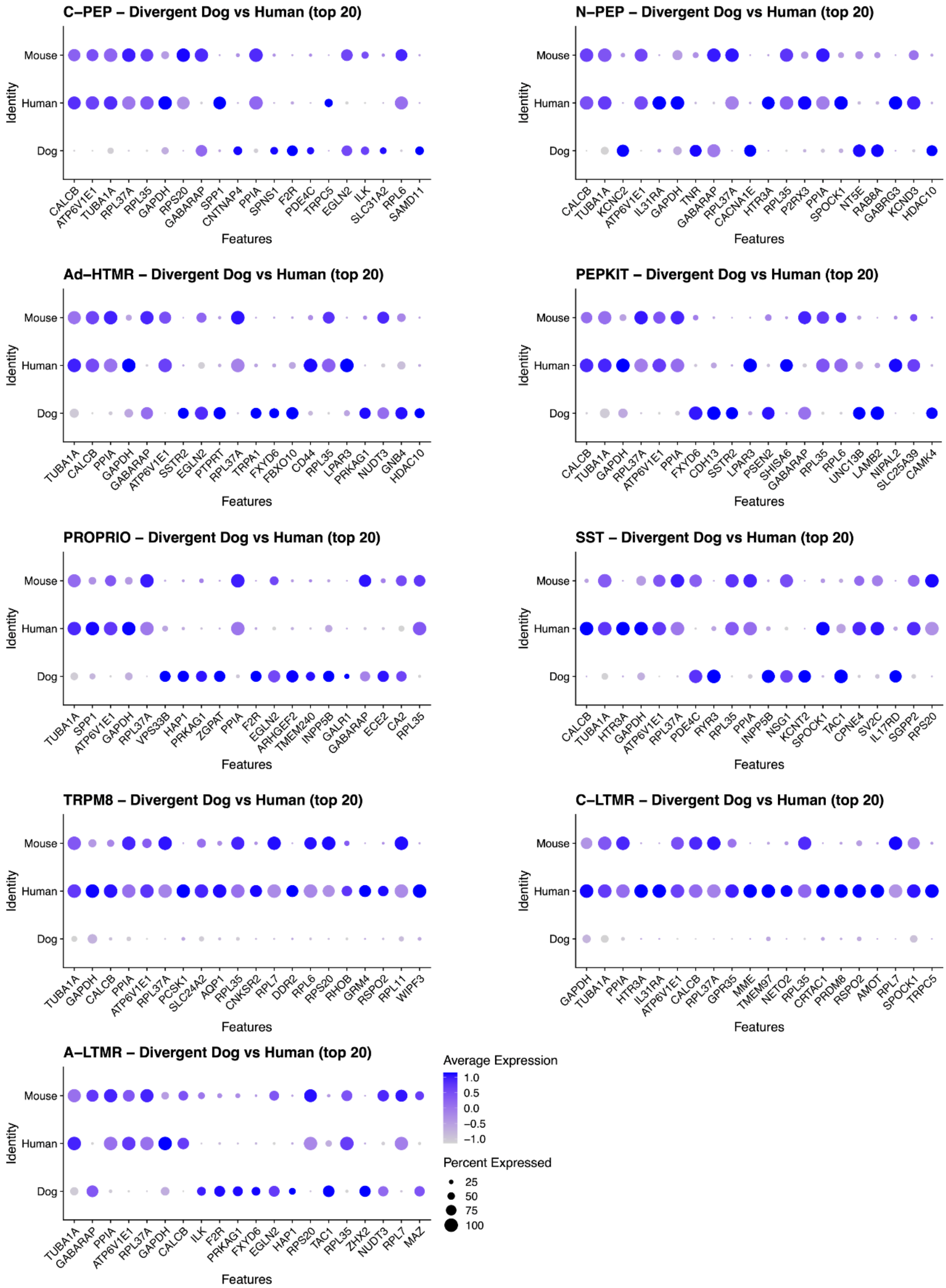

Fig S8. Divergent gene expression between dog and human across unified neuronal clusters. Dot plots showing the top 20 genes with significant species-biased expression between dog and human within each unified cluster; dot colour represents scaled mean expression and dot size the percentage of expressing cells. The top 20 divergent genes were selected by identifying genes significantly enriched in either dog or human ($\log_2 FC \geq 0.25$, ≥10% expressing, adjusted $P < 0.05$) and ranking them by adjusted P value within the species-bias test.

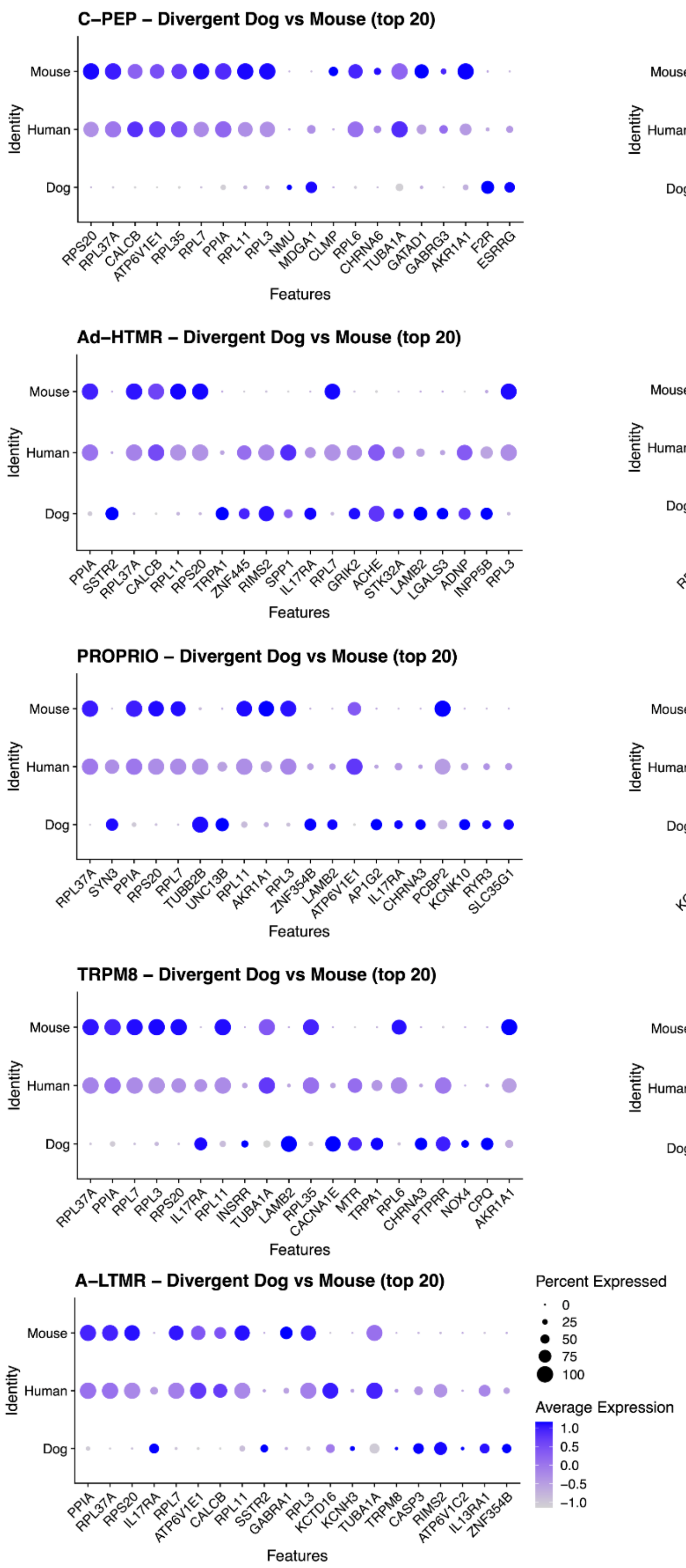

Fig S9. Divergent gene expression between dog and mouse across unified neuronal clusters. Dot plots showing the top 20 genes with significant species-biased expression between dog and mouse within each unified cluster; dot colour represents scaled mean expression and dot size the percentage of expressing cells. The top 20 divergent genes were selected by identifying genes significantly enriched in either dog or mouse ($\log_2 FC \geq 0.25$, ≥10% expressing, adjusted $P < 0.05$) and ranking them by adjusted P value within the species-bias test.

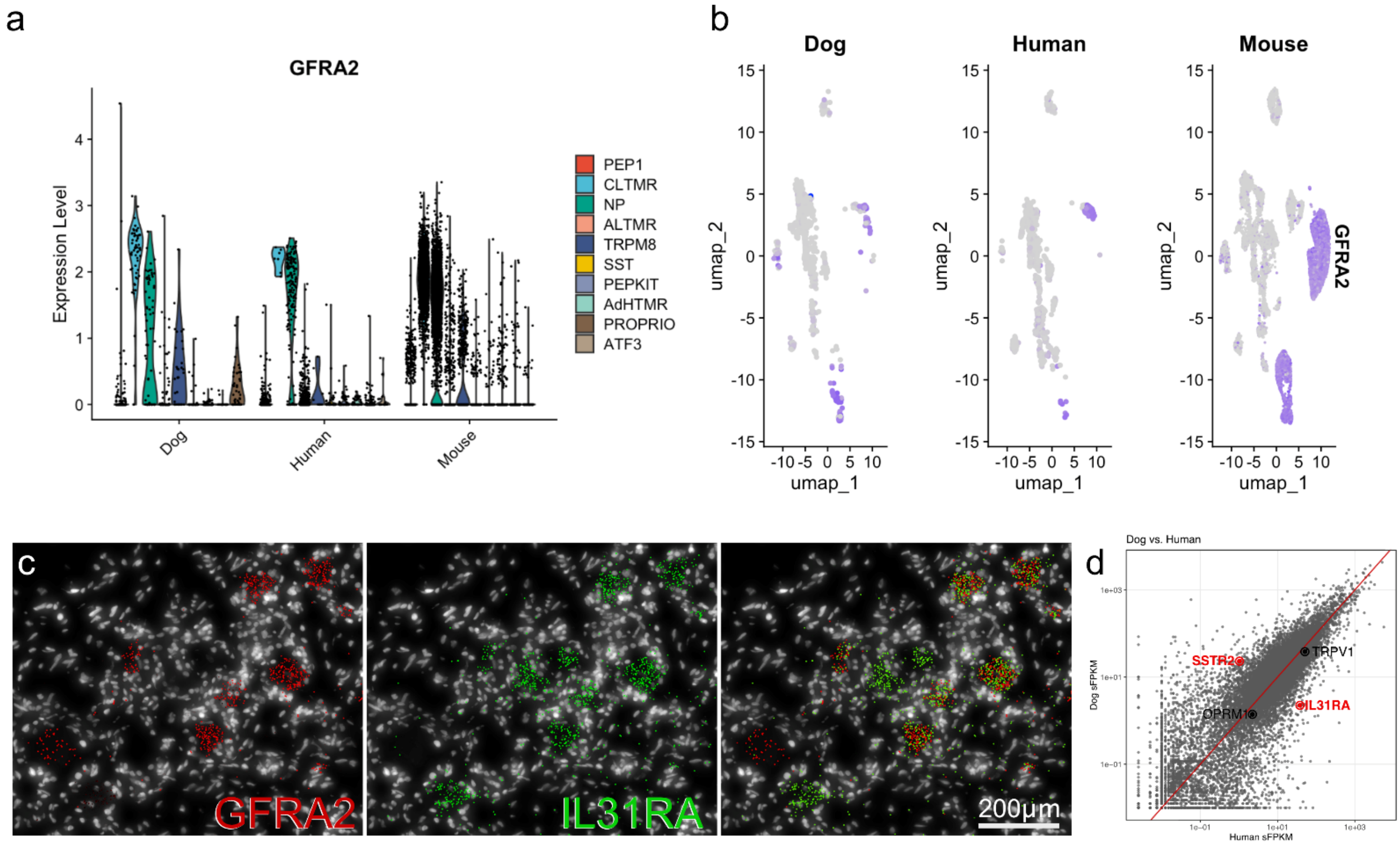

Fig S10. Data to support in situ analyses. (a) Violin plots showing *GFRA2* marker gene expression across unified clusters in dog, human and mouse. **(b)** UMAP feature plots showing the same data and confirming that high *GFRA2* expression occurs in non-canonical peptidergic and C-LTMR clusters and not in SST/OSMR neurons across species. **(c)** Image from Xenium in situ platform using human DRG data from Yu et al. (2024). *GFRA2* and *IL31RA* transcripts are shown in red and green, respectively, with DAPI nuclei in white. Reanalysis of this data demonstrates a significant overlap in cellular expression between the two genes in human DRG tissue. **(d)** Reanalysis of bulk sequencing data from Iadarola et al. (2018) where transcript abundance in human DRG bulk-seq data is compared to that in a high-quality canine dataset. An excellent correlation is evident, but some outliers are seen. On this basis *IL31RA* transcript abundance is lower in canine DRG than in humans whereas *SSTR2* is higher.

mRNA	Gene name	Organism	Nº of binding sites	Probe Channel	Amplifier
IL31RA	Interleukin 31 Receptor A	Dog (*Canis lupus familiaris*)	Up to 20	Channel 3	Alexa Fluor-647
GFRA2	GDNF family receptor alpha 2	Dog (*Canis lupus familiaris*)	Up to 20	Channel 1	Alexa Fluor-546
SSTR2	Somatostatin receptor 2	Dog (*Canis lupus familiaris*)	Up to 20	Channel 2	Alexa Fluor-546
KIT	KIT proto-oncogene receptor tyrosine kinase	Dog (*Canis lupus familiaris*)	Up to 20	Channel 1	Alexa Fluor-647

Table S1. Summary table of HCR™ Gold RNA-FISH probes and amplifiers.

Breed	n	Mean	Median	sd	Min	Max	Q1	Q3
Collie	100	47.8	46.3	15.0	23.5	80.9	35.8	58.7
GSD	100	48.7	49.0	15.2	22.3	81.0	35.7	59.6
Greyhound	100	49.1	49.2	14.1	21.1	81.0	38.2	60.5
XL Bullie	100	44.7	42.0	13.7	21.0	80.4	33.6	54.8

Table S2. Individual dog data used for neuronal size analysis in Figure 1. Data represents diameters in μm.

**Dog ID**	**Dog Breed**	**Age (year, y; months, m)**	**Sex**	**RIN (if applicable)**	**Technique**
Greyhound	Lurcher X	2y	Male		IHC
German shepherd	German shepherd	2y	Male		IHC
Collie	Collie X	2y	Male		IHC
Bulldog	Bulldog X	2y	Male		IHC
1001	Beagle	1y	Male	9.3	FLASH-seq
1003	Beagle	1y	Male	10	FLASH-seq
Male 1002	Beagle	1y	Male	9.1	FLASH-seq
Male 4	Beagle	2y	Male	9.3	FLASH-seq
Male 5	Beagle	2y	Male	9	FLASH-seq
Male 6	Beagle	2y	Male	9.7	FLASH-seq, FISH
Female 1	Beagle	9m	Female	8.8	FLASH-seq
Female 2	Beagle	9m	Female	7.8	FLASH-seq
Female 3	Beagle	1y	Female	8.4	FLASH-seq
Female 4	Beagle	1y	Female	8.7	FLASH-seq
Male1003	Beagle	9m	Male		FISH
FC1	Beagle	1y	Female		FISH
F2	Beagle	10m	Female		FISH

Table S3. Individual information for each dog: Dog ID, breed, sex, age, use (immunohistochemistry, IHC; in situ hybridization, ISH) and RNA Integrity Score (RIN).

For Table S4 see additional spreadsheet

	Number Small (<40µm) Neurons	Percentage of Small (<40µm) Neurons	Number Medium (40-60µm) Neurons	Percentage of Medium (40-60µm) Neurons	Number Large (>60µm) Neurons	Percentage of Large(>60µm) Neurons
**SSTR2**						
SSTR2-	76.25 (65 - 107)	57% (49% - 67%)	59 (40 - 76)	54% (44% - 69%)	20.25 (9 - 32)	72% (53% - 79%)
SSTR2+	38.25 (33 - 46)	29% (27% - 30%)	26 (19 - 30)	24% (20% - 33%)	4.25 (2 - 7)	15% (8% - 29%)
SSTR2++	18.75 (6 - 30)	14% (4% - 22%)	23.5 (8 - 41)	22% (7% - 28%)	3.5 (2 - 5)	13% (9% - 18%)
**KIT**						
KIT-	51.75 (33 - 72)	39% (28% - 45%)	88 (69 - 112)	81% (77% - 92%)	27.75 (14 - 42)	99% (98% - 100%)
KIT+	38.75 (30 - 48)	29% (25% - 41%)	12.5 (3 - 20)	12% (3% - 16%)	0.25 (0 - 1)	1% (0% - 2%)
KIT++	42.75 (35 - 52)	32% (28% - 38%)	8 (5 - 14)	7% (6% - 10%)	0	0%

Table S5. Neuronal counts (shown as mean and range) for SSTR2 and KIT categories per neuronal size class. Data is from 4 animals. Neuronal diameter categories grouped in small (<40 µm), medium (40–60 µm) and large (>60 µm). Data used for Figure 4.

Table S6. Full list of genes from domestication studies overlapping with cluster DEGs

Cluster	Gene
A-Beta LTMR	NTNG1, ZACN, DEFB119, ZMAT4, TMEM26, GALR2, SSH2, BTAF1, CEP135, TMC8, UNC13B, CACNA1A, BEND5, C5AR1, EXOC7, FHOD3, TIAM1, FSTL4, KIAA0586, FANCG, ZNF516, PDE7B, SEMA4A, POLR1E, DNAH17, ARHGEF39, KIAA1328, COPS7B, SCN2A, ACAD11, REEP3, ACOX1, DNMBP, IDE, PIGO, MAPKAPK3, LMBR1L, LYST, DNAH3, PLOD2, ACSM5, ZNF236, TTC39A, RYR3, DUT, NRG2, ELF2, CYFIP1, RALGAPA2, SBF2, SUGP1, PCSK4, MARK3, CADM2, TNKS2, BACH2, ADM, CAMKMT, ITCH, HECW2, ZZEF1, TMEM120B, PIK3R1, ATXN2, POMT1, ART3, MDM2, FKBP11, XYLB, SF3B1, LARS2, CNTN5, MAU2, NUP54, ARHGAP32, LRRTM3, BAZ2B, SI, VEZT, DIP2C, LARP1B, LRIG3, RNPC3, AHCYL2, NUP107, USP15, TUBGCP5, NR2C1, CNOT2, HYDIN, TRMT1, SCYL3, MTMR4, AFMID, STX10, DNAL1, ZNF396, CELF1, RFX3, AQP11, NPHP3, ROR1, PLXDC2, MYCBP2, PLEKHH2, STK10, CALB2, TOM1L2, FGD6, LMOD1, RFXANK, CMTR2, RHOF, MON2, ZNF24, ACVR1, ZNF407, PIGL, PIK3C3, GBF1, ZNF23, POLN, HTR2B, MAP6, HERC2, MFSD8, GALR1, CCNT1, PRKN, ST7, CPEB3, ASIP, PMM2, ARID1B, CNDP1, EHHADH, PAQR6, TRDN, CEP78, MBD2, SEPTIN4, RANBP17, PPM1H, PSD2, POLI, COL11A1, ADCY8, WDR26, RMDN2, KXD1, IKZF1, MET, ANGPTL7, SMCHD1, NRXN3, KLF12, RAB3GAP1, ADCY6, FRG1, NAT8L
A-PEP	ZMAT4, NAT8L, NGFR, HAPLN4, SEMA3D, ADM, ADCY8, RNF6, GALR1
C-LTMR	RTBDN, DAPL1, FRMD6, TLX3, SMO, FGF1, CACNB3, ABHD12, BACH2, CRYM, GALR1, SAMD12, NEDD4L, C3orf18, TSPAN14, GDPD5, KCNC2, MAPKAPK3, LIMCH1, METTL22, LMNA, REEP6, CUX2, RALY, GRB10, CISH, NAV1, ZC3H12A, CNTN5, ADAMTSL5, DOCK2, TBC1D22A, PSAT1, GAD2, METAP2, TIAM1, PCSK4, ASAP1, NTAN1, CTH, BDH2, PLCXD3, PRMT2, PLEKHA4, MEIS3, RFXANK, ACVR2B, GLIS1, CEP78, ACOX1
C-PEP TRPA1+	MYOF, FBN1, NCAM2, GPR139, SCUBE1, PLCXD3, FOXP2, NEDD4L, KCNJ3, PRRC2B, FAM107B, CYP1B1, PCDHGC5, PRKACB, FKBP8, PYGB, PNMA1, RIMS2, PRMT2, TLN1, CARHSP1, YWHAH, DIP2C, CACNB3, ACOT6, EEF1A1, HSPD1, ELAVL4, CNDP2, EPS15, CCSER2, SLCO2B1, SUSD6, PSAT1, COL11A1, GNAQ, RFTN2, GDPD5, PEMT, TSPAN14, MOB4, MTOR, HSD17B12, RPS15, PRDX3, CCT3, GNA12, SNX19, ZFYVE28, RNF103, SERPINH1, NKAIN2, ANKRD44, TMEM59L, SNRPN, SETD9, PCDH18, PLEKHM3, TOMM20L, ZMYND11, NPM1, OPRM1, SRP72, PGRMC2, ATG13, ASAH1, GRB10, CETN3, ACTR10, ABHD12, LRPPRC, PSMD1, ACSS2, FBXL3, TLX3, RRN3, SLC25A44, ARRB1, VCP, GTF2E2, ARL2BP, PARP12, SDAD1, UCK1, NINL, CUEDC1, NIPA2, RPS3, PRNP, COQ10B, CALR, CUX2, BCAT2, HARBI1, GNL2, PPM1H, HPD, NCAPG, ARID4A, PSD4, REEP1, SMIM15, FARSA, AMBRA1, CREB3, NAT8L, DDX23, MRPS23, MARS2, DHX9, KAT7, PDXDC1, LMNA, MANEAL, METAP2, NTAN1, ITGB1, HIPK2, RNF6, PGS1, TVP23B, ARID1B, SNIP1, PPM1E, RAD23A, FRMPD1, SMOX, DIAPH1, SMCHD1, PIK3C3, POLI, STARD6, HINT2, CNIH4, CLN5, TBC1D22A
C-PEP.CHRM1	FBN1, NCOR2, TOPORS, FRG1, BAZ2B, DNASE2, SRSF11, RYR3, NRXN3, COL11A1, SCN2A, UNC13B, ACVR2B, ELF2, RNPC3, ARID4A, CDC42BPA, MED13, MYCBP2, NCOA6, GPR139, HERC2, GDPD5, IKZF1
C-PEP.GAL	CLIC4, FKBP8, FLVCR2, EEF1A1, HINT2, SCUBE1, GTF2E2, MOB4, PRDX3, RPS15, UCK1, PNMA1, YWHAH, TLN1, CNN3, OPRM1, GNAQ, SYN2, PCDH18, ACTR10, PSMD1, PCDHGC5, RIMS2, NCAM2, EPS15, CARHSP1, PRRC2B, HSD17B12, NPM1, HSPD1, PLEKHM3, PRMT2, NGFR, LCORL, RPS3, COQ10B, DNAJC15, ZMYND11, ABAT, LRPPRC, UBA52, PLCXD3, CCT3, ARRB1, NIPA2, FBXL3, CCDC107, GADD45GIP1, ELAVL4, CUL1, ASAH1, MANEAL, RNF103, PGRMC2, PTPMT1, ACOT6, CNDP2, REEP1, NXPH3, CAPS2, PYGB, KAT7, GNA12, RBPMS, CETN3, SETD9, RAP1B, SNX19, PEMT, PDE6D, PACRG, VCP, NCAPG, GCDH, RAD23A, SYNGR2, CREB3, ARL2BP, RNF6, LMNA, EHHADH, FXR1, PRKACB, SNRPN, STARD6, SLC35B1, KCNJ3, PAK1, SRP68, SPOP, TMEM59L, GLMP, EIF2S2, PPM1E, FRMPD1, CNIH4, SLC44A3, MXD4, CALR, SLC25A44, TLX3, CISH, SRP72, RAI1, BCAT2, ATG13, DNAJC19, FARSA, TRMT61A, ZFYVE28, UBA5, NUBPL, AGAP1, TBC1D22A, NKAIN2, ARID4A, DDX23, MRPS23, TVP23B, PSD4, SLK, GBA2, RFTN2, PSAT1, COMMD10, HPD, CUEDC1, RSPRY1, EXOC7, SMIM15, PDXDC1, COG6, METAP2, UBIAD1, RANBP17, PGS1, CCSER2, MAP6, LRRN3, KBTBD4, CACNB3, RAD51C, ANKS4B, NOCT, RCN2, GPR139, TMEM167A, ITGB1, CELF1, ABHD12, DCAF10, ACSS2, NACC1, CNOT4, FAM169A, EXOSC3, MEAF6, DHX9, IPO9, FAM117A, GNL2, SUSD6, TPGS2, NR2C2AP, JRKL, ARMC9, AMBRA1, RND1, FBXW11, NVL, HARBI1, NUP107, GLIPR1L2, RRN3, KXD1, HERC2, CCNT1, NOL8, RHOF, EXOSC10, CYP1B1, ST7, METTL22, ALDH18A1, SERPINH1, CTH, DGAT2, NDUFAF2, NUP54, CCDC65, CA9, CERK, GLIPR1, MARS2, MFSD8, MAST1, NFKB2, TRMT10B, REEP6, ACMSD, NDUFS3, FAM107B, BDH2, OMA1, SMCO4, C4orf33, CCDC177
C-PEP.TRKB	TCF4, LRPPRC, OPRM1, EEF1A1, CARHSP1, BOLL, RCN2, CNN3, GAD2, PIK3R1, PARP12, NGFR, TRMT61A, LRRN3, TVP23B, RRN3, SUSD6, AGAP1, PLEKHM3, TMEM59L, GNA12, SLCO2B1, NPM1, HIPK2, GCDH, ACSS2, CALR, GNAQ, CNDP2, PGRMC2, DHX9, SLC45A4, REEP1, TUBGCP5, TLN1, RPS3, SLC25A44, EPS15, EHHADH, CCNT2, ELL, EFCAB5, SNIP1, GNL2, TMEM242, FAM169A, DIS3L2, NTAN1, NIPA2, LGR5, MTOR, PGS1, ACVR1, IPO9, ST7, ARL2BP, GLMP, NOCT, SLK, ZNF786, ROR1
C-PEP0	RALY, RGP1, RPS3, GADD45GIP1, C3orf18, CREB3, SMYD3, MBD2, SEPTIN4, CENPV, SCYL3, ARHGAP32, XRCC4, PEMT, RPS15, XYLB, MIER3, IER2, RRM2, POLN, DDC, C18orf54, TNFRSF11B, ZNF396, TEX14, C5AR1, TCTN3, CLIC4, LGR5, IKZF1, FGD6, HSF5, ART3, PITX1, TMEM79, CLEC5A
N-PEP.MRGPRD/GFRA2	HYDIN, DGKI, STXBP6, KCNC2, NKX2-8, PLCE1, TSPAN14, DDC, NAV1, PDE7B, CENPW, GRB10, SH2D4B, NEDD4L, ANKRD44, GDPD5, PLEKHH2, PRNP, ZMAT4, FHOD3, BACH2, RALY, ABHD12, SAMD12, CARHSP1, ASAP1, RYR3, BDH2, PRKCA, TMEM132D, GLIS1, MGARP, TEN1, KIFAP3, EML6, LRRN3, CISH, ALDH18A1, DIP2C, RAD23A, TK1, MGAM, CUX2, METAP2, GNAQ, CCSER2, HECW2, ACAD11, STX10, CEP78
PEP-KIT	NFIX, GPR20, MYCN, CACNA1A, NRXN3, LRRTM3, IER2, MEIS3, OPRM1, SLC44A3, ITGB1, RBPMS, EML6, DNMBP, RMDN2, PAK1, TCF4, ZMAT4, PCDH18, MRC2, PYGB, PARP12, KIFAP3, ASAP1, ELL, ATXN2, NGFR, GBF1, ACSS2, FAM117A, TNKS2, FHOD3, PRKCA, POMT1, FRMPD1, TMC8, NCAN, BEND5, PPP1R15A, CLEC5A, KIAA1328, MYCBP2, TMEM59L, ELAVL4, CYFIP1, CLN5

	, NPR2, C5AR1, FXR1, TPM2, XYLB, ARMC9, TOM1L2, SCLT1, RNF6, ZP2, NRF1, SCUBE1, ARID4A, AQP11, FBXW11, RAB3GAP1, UCK1, DGKI, IDE, EXOSC3, POLR1E, IPO9, FBXO10, SI, COPS7B, EFCAB5, NR2C1, REEP3, LARP1B, ITCH, CCNT2, UNC13B, CALR, RAP1B, SLC9B2, LGR5, ANKS4B, SBF2, TEN1, UBA5, WDR26, MEAF6, LYST, CEP135, ZNF516, TTC39A, GLIPR1, TLN1, EXOSC10, SUGP1, CERK, NCOA6, C18orf54, POLI
PROPRIO	HAPLN4, CALB2, ADCY8, MET, MYL2, ADM, CRYM, SEMA4A, NTNG1, DEFB119, FSTL4, FHOD3, SH3GL2, C3orf18, LRRTM3, ACOX1, MAPKAPK3, CADM2, DNAH17, TPM2, MYOF, CNTN5, SATB2, NAT8L, PIK3R1, SLC45A4, SEPTIN4, AHCYL2, TIAM1, MBP, GALR1, PAK1, FGF1, RMDN2, SMO, HECW2, OMA1, ARHGEF39, DUT, CCSER2, ARL9, FANCG, RAB3GAP1, KCTD12, TOM1L2, BEND5, PCSK4, DYNC2LI1, CUX2, TMEM59L, PIK3C3, MTMR4, SMG5, IDE, ST7, SCYL3, ACSM5, CNN3, PPM1H, VCP, GLRA1, TMEM242, MGARP, GPR139, CCNJ, COL11A1, KIFAP3, ARL2BP, SSH2, POLR1E
SST	IL31RA, PLCE1, STXBP6, VWC2, RYR3, ELL, TIAM1, NAV1, FGF1, CEP78, GPR139, TCF4, PPM1H, CENPW, HYDIN, RAP1B, NOL8, DGKI, CARHSP1, RIMS2, CYP1B1, MEIS3, ASAP1, PLCXD3, KCNJ3, MAPKAPK3, FAM180B, CDC42BPA, EML6, FRMPD1, GRB10, GDPD5, GLIS1, HECW2, FHOD3
TRPM8	FOXP2, LGR5, HIPK2, ANKRD44, CALB2, ACSS2, PLCXD3, SCAPER, SNRPN, CHRM5, HEMK1, NKAIN2
